# Socioeconomic and psychosocial determinants of substance misuse – a national perspective

**DOI:** 10.1007/s12024-023-00702-6

**Published:** 2023-09-08

**Authors:** Lilli Stephenson, Corinna Van Den Heuvel, Roger W. Byard

**Affiliations:** 1 School of Biomedicine, Level 2 Helen Mayo North, The University of Adelaide, Frome Road, Adelaide, 5005 SA Australia; 2 Forensic Science SA (FSSA), Adelaide, South Australia 5000 Australia

**Keywords:** Illicit, Drug, Trends, Forensic pathology, Forensic science, Toxicology

## Abstract

Accurate estimates of population drug use and an understanding of the factors that influence substance choice are essential for the development of appropriate and targeted prevention strategies and campaigns. This review aims to provide an overview of the socioeconomic and psychosocial factors that influence substance use patterns within the Australian population through exploration of current and historical examples of substance misuse. Australia’s comparatively large online drug market is reflective of the country’s relative geographic isolation and high local drug prices. Legislation, particularly relating to cannabis cultivation and personal use, has evolved significantly in response to increased scientific and commercial applications and changing attitudes towards medical and personal use. Methylamphetamine use is disproportionately high, attributed to Australia’s geographic location, high rates of local manufacture, steady cost, and increased purity. Despite the increased cost of cocaine over time, the profile of users appears to dictate rates of use. The prevalence of injecting drug use is driven by a lack of education, perceived risk, stigma, and other social factors. Additionally, psychosocial factors also contribute to substance misuse among specific population subgroups such as petrol sniffing among Indigenous Australians. Understanding the reasons for geographical variability in illicit drug use assists in the interpretation of substance-associated behavior in specific groups/populations and in guiding future intervention efforts and predictions of emerging trends. In addition, an understanding of factors influencing local drug usage may assist forensic practitioners in evaluating the occurrence and effects of particular substances that may emerge as significant factors in drug-related deaths.

## Introduction

Globally, drug misuse is a major contributor to disease, illness, injury, and death. In 2017, drug misuse was responsible for the loss of approximately 42 million years of “healthy” life and 585,000 deaths [1], with over 35 million people diagnosed with a drug use disorder in 2018 [1–3]. An illicit drug is defined as a substance for which possession, use, production, importation, and distribution is prohibited by law [4]. Illicit drug use contributes significantly to total drug use, including both the illegal use of prescription medications and that of prohibited substances [3]. Over the last 10 years, illicit drug use has increased by 28% globally, with approximately 269 million past-year drug users in 2018 [2, 3].

In 2018, illicit drug use also contributed to a significant proportion of the total burden of disease and deaths in Australia (3% and 1.8%, respectively), driven by trends primarily among young people, and the use of opioids [5]. In terms of drug use trends among the Australian population, the 2019 National Drug Strategy Household Survey (NDSHS) found that cocaine use was at its highest level in the last 20 years but rates of substance use in general were decreasing among younger generations [6]. It also appears that attitudes towards drug use are changing, with more Australians supportive of cannabis use and pill-testing than in previous reporting periods [6]. Changes to drug scheduling (e.g., codeine being made a prescription-only medication in 2018) also contributed to a decrease in extra-medical use of pharmaceutical substances, particularly pain killers and opioids [6]. The term “drug” implies use of a pharmaceutical substance in the context of medical or extra-medical use. However, there is a much more diverse range of substances, both pharmaceutical and non-pharmaceutical, that may be abused within given populations and not captured by the primary literature. These will be explored in further detail with illustrative examples.

While there is extensive research on substance use and health-related outcomes in particular populations, analysis of socioeconomic and psychosocial factors that influence drug choice and impact upon drug prevalence within specific communities is comparatively limited. Significant factors which influence substance choice include availability, geographic affluence, substance policies, law enforcement activity, targeting, product formulations and quality, cost, demographics, drug-related stigma, perceived risk of harm, and traditional use [7–10]. There are many examples of idiosyncratic substance use in countries and demographic groups which can be attributed to the intersectional relationship between and among many of these factors [7, 11–13]. An understanding of these complex and inter-related issues is essential for the development of targeted prevention strategies and may assist in predicting trends in future substance use with reduced associated morbidity and mortality. This review provides an overview of these factors in the context of the Australian population and explore current and historical examples of substance misuse that are particularly prevalent within specific demographic groups or geographic areas. A literature search was undertaken for reports of substance misuse both from the internet in general and from specific search engines such as PubMed and Google Scholar. In addition, relevant media articles and national reports from various health and government bodies were also evaluated.

## Availability

As the availability of drugs fluctuates, trends in drug use vary accordingly. In this context, an economic theory known as the “substitution effect” explains how drug preference is primarily driven by availability, alterations in price and/or an individual’s income [7]. For example, someone whose main drug of preference is heroin may substitute this for morphine if it is deemed to be more widely available or cheaper by comparison. This scenario may also occur in the opposite direction with changes to local drug prices and drug availability, particularly as ‘doctor shopping’ (i.e. obtaining several prescriptions by visiting multiple doctors’ practices) has become a more widely recognized problem [14]. Availability of drugs may be influenced by several other factors including means of access, social dynamics, geography, legislation, and large-scale supply interventions [8, 15, 16].

Relative geographic isolation, both national and regional, may also influence the ability to obtain certain drugs, as is demonstrated by significant regional and sub-regional differences in drug use prevalence for specific substances. For example, in the Oceanic region, the number of past-year cannabis, cocaine, amphetamine, and ecstasy users are amongst the highest in the world, while the number of opioid users is comparatively low [17].

Before the advent of the internet, buying and selling illicit drugs was a more intensive undertaking often associated with significant personal risk. However, the “dark net,” among other online platforms, has revolutionized the modern drug market increasing the availability and access to drugs by overcoming the limitation of geographic isolation [18] and reducing the perceived risk of detection [19]. The dark net is a network within the internet that is only accessible with specific software or authorization and may be used for both illegal (e.g., illicit drug purchasing) and legal activities [20]. One of the first “dark net” drug markets (crypto markets) was created in 2006 and was live up until 2012, when it was closed after a two-year investigation by the Federal Drugs Administration (FDA). The website served as a marketplace for a variety of illicit substances for thousands of users in 34 different countries [21]. However, although it would seem more convenient, it appears that only a small proportion of younger drug users actually choose to purchase their drugs through the “dark net” [10], with most preferring to obtain drugs from friends [19].

### Online drug purchasing in Australia

In Australia, online drug markets have rapidly grown in popularity, as new iterations of previous websites emerge. In the most recent 12-month reporting period, there was a considerably rapid turnover of crypto market platforms [22]. The comparatively high number of online drug dealers in Australia compared to other countries is thought to reflect the country’s relative geographic isolation and high local drug prices [23]. While the online Australian drug market is largely comprised of Australian sellers and Australian buyers which is largely attributed to the reduced risk of border seizures (and legal penalties) in addition to better-quality products [23], individuals may also buy drugs online from overseas. Importation methods can include international mail, sea or air cargo, and air passengers or crew [24].

Between October 2021 and September 2022, the most common substances on all crypto market listings were cannabis (30%), followed by cocaine (7.6%), benzodiazepines (7.5%), MDMA (7.5%), meth/amphetamines (6.1%), and opioids (6.1%) [22]. However, opioids showed the highest rate of growth concomitant to a decrease in LSD listings in comparison to other substances [22].

Use of online resources often depends on social network dynamics and the drug use habits of acquaintances, friends, and family. However, the digital age has also introduced other newer, more popular platforms in high socioeconomic countries (including Australia), which are used to facilitate the purchase of drugs; these have included Snapchat, Instagram, and WhatsApp [19, 25]. These platforms provide quick, easy, and convenient means of facilitating buyer–seller interactions and as they are perceived to be more secure than traditional methods, they are rapidly growing in popularity [19, 25].

## Legislation

Legislation (i.e., regulation and prohibition) is one of the main strategies employed to reduce drug use in many countries [8]. At a global level, drug legislation has needed to evolve to target not only traditional plant-based drugs (e.g., heroin, cocaine, and cannabis), but also the emergence of new psychoactive substances (NPS) and the illicit use of pharmaceutical drugs such as opioids [26]. One of the current difficulties in regulating illicit drugs, particularly NPS, is in keeping drug policy abreast of rapidly changing drug use patterns.

Over the last 20 years, integration of national drug policies underpinned by harm reduction strategies has become increasingly popular, supporting larger-scale interventions to reduce supply and demand [16]. However, legislation varies widely between and within countries. For example, Australia’s drug policies historically have been considered relatively strict, often applying penalties for personal use of illicit drugs [27]. Responses from the 2014 Global Drug Survey indicate that residents from countries with prohibition-based drug policies such as Australia, would feel more confident in utilizing harm-minimization programs if drug policies were to be liberalized, due to reduced fear of criminal charges [27]; i.e., some Australian residents support interventions underpinned by education and treatment, rather than enhanced law enforcement strategies [28]. There is also growing evidence to support decriminalization of drug use such as low rates of drug use in European countries where this has occurred for personal drug use [29].

There is disagreement concerning the measurable impact of drug policy and legislation on the prevalence of drug use, as quite similar drug trends have been observed between counties with markedly different drug policies and vice versa. For example, the United States (US), United Kingdom (UK), Netherlands, Switzerland, and Australia all have very different drug policies but present similar trends in certain, but not all, drug categories [16].

While the issue of drug policy is multifaceted, it is evident that policy reform has the capacity to influence drug use patterns, and subsequently reduce morbidity and mortality. Conversely, it has been argued that changes in drug policy may not directly correlate to reduced drug use or positive outcomes for drug users. Thus, it remains unclear to what extent legal penalties influence an individual’s choice to purchase and use drugs.

### Cannabis legislation

In 2020, cannabis use among adults in Australia and New Zealand was found to be significantly higher than the global average [30]. The popularity of cannabis use in Australia may be attributed to stability in the cost, purity, and accessibility of cannabis compared to other illicit drugs. Legislative reform relating to industrial and commercial hemp cultivation in several Australian states in the early 2000’s could be considered the catalyst to a change in attitudes towards the legalization of cannabis for medical and personal use. While drug policies related to personal cannabis use in Australia have historically followed a strict, no-tolerance approach, recent developments have seen the legalization of commercial cannabis growing for medical and scientific purposes. Furthermore, states and territories including the Australian Capital Territory have also legalized small amounts of cannabis for personal use without penalty, in line with changing national attitudes. The 2019 NDSHS found that 41% of Australian’s surveyed support the legalization of cannabis for personal use, which has nearly doubled since 2016 [6].

More recent studies using survey data indicate, however, that the legalization of cannabis has been associated with increased prevalence of use and substance use disorders, allowing for the lag time between the passage of laws and the development of trends [31–34]. Furthermore, these studies have highlighted that increased cannabis use has occurred not only among frequent users, but also among previously non-using adolescents and young adults [6, 31, 32]. Another important issue following increased availability due to legalization has been a decrease in the perceived risk of harm [34–37].

## Product formulations and quality

Evolution and optimization of drug manufacturing processes has resulted in a rapid increase in the number and variety of drugs available on the market, particularly involving NPS. Population drug use trends often reflect a desire to obtain these newer, more popular drugs as higher quality formulations of traditional drugs enter the market. For example, ecstasy was a popular recreational drug in the US in the late 1970’s until it was listed as a Schedule I drug in 1985 in response to increasing concerns about abuse potential [38, 39]. Australia also criminalized the possession and use of ecstasy [40]. However, ecstasy use has recently re-emerged, partly in response to increased availability of higher quality products with newly available formulations (powder and crystal) [2]. A study of illicit drug substitution among high-risk drug users found a preference for substituting traditional drugs with either newer drugs, including NPS [41]. The most common substitutions were those within the same drug class, where the substituted drug elicits similar physiological effects (e.g., heroin for methadone, cannabis for synthetic cannabinoid receptor agonists (SCRA’s)) [41]. SCRA’s are potent synthetic compounds that bind with the same receptors as endogenous cannabinoids (CB_1_ and CB_2_) [42]. However, synthetic cannabinoids are complex structures, allowing for a potentially endless number of chemical modifications to create new products with unknown potency and physiological effects [42]. These substitutions may also involve more complex factors than the desire for new product formulations with improved quality, but also relationships between availability, cost, drug policy and desired effects. Enhanced domestic drug manufacturing processes have also increased the local availability of specific drugs such as cannabis and methamphetamine.

### Domestic manufacture of methylamphetamine

In 1998, an increase in the number of fatalities associated with amphetamine derivatives was identified in Adelaide, South Australia [43] and 20 years later, Adelaide was named the “methamphetamine capital of the world” based on a seven-year wastewater study [44, 45]. The wastewater study involved daily sampling of wastewater over a period of one week between 2011 and 2017 and analysis for the presence of popular drugs of abuse (e.g., cocaine, methamphetamine, and MDMA) by liquid chromatography-tandem mass spectrometry [44]. Although use by people aged 40 years and over appears to be increasing [46], meth/amphetamine consumption among younger age groups (20–29 years) is declining [6]. It is unclear whether increasing use among adults over the age of 40 is due to the aging of long-term methylamphetamine users or an increasing appreciation by younger people of the risks associated with the drug. Other factors that may have contributed to reduced rates of use among young people include availability or popularity of other drugs and their associated effects, changes to social landscapes, and group dynamics.

The Australian market for methylamphetamine is relatively stable according to the 2017–2018 Illicit Drug Data Report, where amphetamine-type substances (ATS) constituted nearly 40% of national illicit drug seizures, only surpassed by cannabis [47]. However, since 2014–2015 when ATS border detections were the highest on record, the number of methylamphetamine detections have consistently decreased [48]. The reduction in numbers of detections may be partially due to the significant increase in domestic production of methylamphetamine. Compared to other countries, Australia has a comparatively high number of clandestine laboratories and thus, a large domestic market for methylamphetamine [23]. The majority of clandestine laboratories detected in Australia in 2017–2018 were producing methylamphetamine, most of which were residential facilities operated by users/addicts [47]. In terms of geographic variability, both recent and lifetime methamphetamine use has been found to be higher in rural areas compared to urban regions [49]. This may support the hypothesis that drug use is being driven by the presence of methylamphetamine manufacturing sites in rural areas and/or urban populations demonstrating a preference for alternative drugs.

**Fig. 1:**
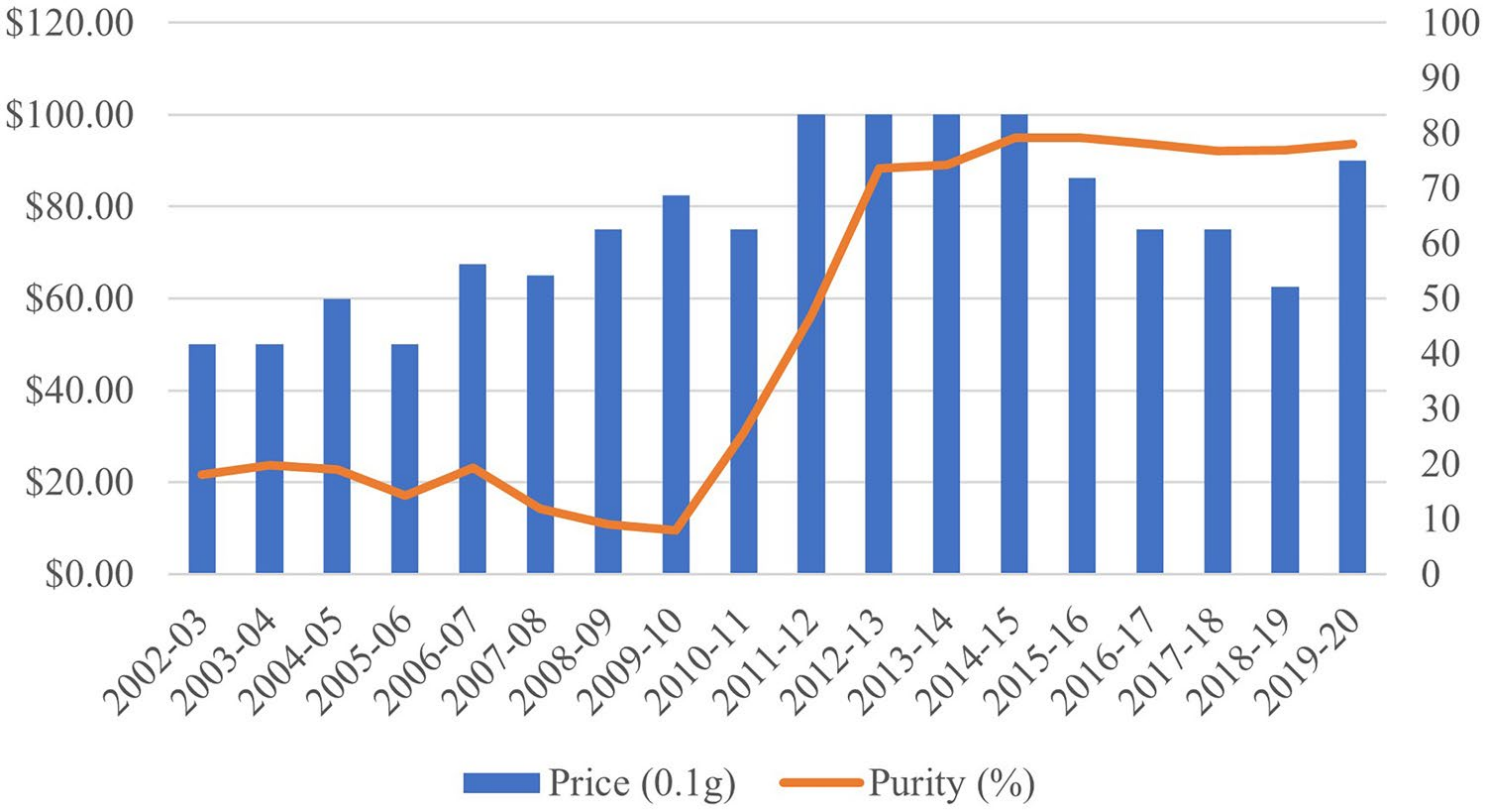
Median methylamphetamine price and purity in Australia between 2002–2003 and 2019–2020 (adapted from [4, 47, 48, 54, 55, 87–99])

Although there are various forms of ATS available on the market, crystal methamphetamine (crystal/ice) remains the most popular [6]. This is confirmed by survey data from a sample of people who inject drugs in several Australian states between 2000 and 2022 who report that crystal methamphetamine use has steadily increased to comprise approximately 80% of responses, while base and powder forms have decreased to comprise less than 10% of responses, respectively [50]. The “advantages” to using crystal methamphetamine over other forms include a generally higher purity and more intense “high” [6]. Over the last decade, the price of “street” methylamphetamine in Australia has stayed relatively stable, while drug purity has increased significantly [47] (Fig. 1). The appeal of methylamphetamine and ATS in Australia appears to involve a relationship between geographic location, availability through local manufacturing, and a steady cost despite increased product quality.

## Cost

Cost is a significant factor influencing drug prevalence and preference [7]. In a cohort of “dark net” purchasers, 38% of users cited cheaper online prices as the major motivating factor for purchasing drugs compared to buying on the street [51]. Cost has also been cited as a significant factor driving growth within the illicit fentanyl market, as it is cheaper to produce than heroin [52]. However, this is highly variable between socioeconomic and geographic areas. In two cities in Tajikistan, heroin was cheaper than a bottle of vodka and so it subsequently became a popular drug of choice among people who injected drugs (PWID) [12]. High drug costs can also promote risky behaviors to obtain drugs or the necessary purchasing funds. For example, a Canadian cohort of female sex workers had a preference for heroin, despite it costing twice as much as cocaine, and were prepared to actively participate in high-risk activities to support their drug preference [53].

### Changes in drug prices over time

According to data from the Australian Criminal Intelligence Commission (ACIC), all drugs analyzed have increased in price, except for MDMA (Table 1). As discussed above, the price of methylamphetamine has increased in parallel with a significant increase in purity (Fig. 1). The higher price of methylamphetamine may also be contributing to decreasing use among younger people, where rates of use among those over 30 years of age has more or less plateaued [6]. Significant increases in the price of cocaine and heroin were also seen over the four reporting periods. Despite the large increase in price for cocaine, the frequency of use and socioeconomic profile of cocaine users may account for unchanged rates of use (i.e., infrequent use among individuals with a stable income). While heroin has a price comparable to methylamphetamine, rates of use among the Australian population are significantly lower [6]. As discussed, there may be significant stigma attached to injecting drugs, which may discourage uptake in a new, younger cohort of users, in addition to the high monetary cost. The price of hydroponic cannabis (i.e., cannabis grown without soil) stayed relatively consistent over the last 15 years which may partly account for the increased use of cannabis among both regular and first-time users, as well as its wide accessibility [6]. Interestingly, the purity of phenethylamines (mostly MDMA) has markedly increased, yet the price dropped significantly across the four reporting periods. Both recent and lifetime use of MDMA has increased in Australia since 2016 [6].

**Table 1:** Median prices of methylamphetamine, MDMA, cannabis, cocaine and heroin in Australia between 2005–2006 and 2019–2020 (adapted from [24, 48, 54, 55])

	2005–2006	2010–2011	2014–2015	2019–2020	2005–2006 vs. 2019–2020
Methylamphetamine (0.1 g)	$50.00	$75.00	$100.00	$90.00	**↑ **80%
MDMA (1 tablet)	$37.50	$33.25	$35.00	$22.50	**↓ **40%
Cannabis (1 g)	$25.00	$25.00	$23.75	$27.50	**↑ **10%
Cocaine (a cap, 0.2 g)	$47.50	$50.00	$65.00	$80.00	**↑ **68.4%
Heroin (a taste/cap, 0.1–0.3 g)	$52.50	$50.00	$75.00	$85.00	**↑ **61.9%

## Demographics and geographic affluence

Age, sex, and the lifestyle associated with particular demographic groups may influence which drugs are favored. There are also certain age groups that demonstrate higher overall levels of drug taking, in particular youth and adolescents [9], with studies of high school and university students showing higher drug use rates compared to the general population. Potential factors that increase the risk of drug use in adolescents and university students include changes in lifestyle, reduced parental support/influence, and increased stress [56, 57].

Wastewater studies have provided evidence of significant regional differences in drug use; for example, cocaine and methylenedioxymethamphetamine (MDMA) use in Australia is approximately twice as high in urban areas, whereas methamphetamine use is higher in rural areas [49, 58]. Overall, however, drug use is generally higher in urban areas compared to rural areas; a trend observed in many countries [26, 58, 59]. This can be largely attributed to the relationship between urbanization and geographic affluence. An additional consideration may be increased accessibility to drugs in urban areas compared to more remote regions which may also have restricted access through online purchasing (i.e., from poor internet connection and limited postal options).

### Use of cocaine

Studies suggest that cocaine users demonstrate a consistent and specific socioeconomic, educational, and employment profile. The dominant group of cocaine users in Australia appear to be employed, well-educated individuals from high socioeconomic areas with the financial resources to support cocaine use [6, 60]. Between 2001 and 2019, the median age of cocaine users increased from 25 to 28 years of age, suggesting that this was due to the aging of a group of users rather than increased uptake among older persons [6]. Compared to other illicit drugs such as cannabis and meth/amphetamines, cocaine use is less frequent [6]. This may be attributed to the high cost of cocaine ($80 per 0.2 g in 2019–2020) compared to other substances [24]. However, in 2019, cocaine use was found to be at its highest level in the last 18 years among adults [6].

## Stigma and perceived risk

According to a recent survey, the two most significant factors which discourage drug use among young people are the perceived risk of harm and personal disapproval [10]. A cohort of Canadian at-risk youth who recounted their introduction to drug use highlighted a sense of curiosity leading to “nonchalant” choices which seemed inconsequential at the time, only to be made aware of the risks and consequences later in life [13]. While there is a high prevalence of cocaine and ecstasy use in many European countries, the majority of young people consider regular use of these substances to pose a “high” health risk [10]. More than 80% of individuals aged 12 years and older perceived a great risk associated with the weekly use of cocaine, heroin, or LSD (lysergic acid diethylamide) [61]. However, although a majority of young people are aware of the risks associated with illicit drug use, this does not prevent use. For first-time or naïve drug users, initiation or continuation of drug use may be contributed to by limited awareness or a lack of education relating to the risks associated with drug use. Alternatively, perceived risk of harm may not be impactful enough to combat peer pressure within social groups. Conversely, addiction disorders are the most commonly encountered explanation for continuation of drug use among regular drug users despite recognition of the risks, particularly in the setting of injecting drug use.

There are also distinct differences in perceived harm between non-users and users. Compared to users, individuals who do not engage in the non-medical use of prescription stimulants (NMUPS) perceived them to be less safe and more harmful; they would expect guilt, dependence, and anxiety and would be less likely to divert their medication if it were prescribed to them [62]. In general, it appears that those who do not use drugs perceive a higher risk of harm compared to those who do [61, 63].

The main obstacle in disseminating drug-related health information to the general public, particularly youth, is in finding an engaging and influential platform and in determining whether users know and/or care that there is risk involved in such practices. For those aged from 18 to 24 years, the most influential sources of information on drugs are “mine and my friends’ own experiences” and popular culture (books, television, films, famous individual’s activities), but for those aged over 45 years, media reports, expert opinions and drug classifications are more important [64]. For those who already use drugs, the opportunity to discourage drug use may be limited if risk perception is already lowered by familiarity with a particular drug(s) [63]. This may be further compounded by additional factors such as peer pressure, normalization, situational stress, and physical addiction.

### Risk perception and stigma among people who inject heroin

Between 2000 and 2022, heroin and methamphetamine remained the two most prevalent drugs among a sample of people who inject drugs (PWID) from all Australian states and territories [50]. Over this period, rates of heroin use have remained stable, while methamphetamine use has consistently increased in popularity to overtake heroin within the last 2 years [50]. Heroin use is highly stigmatized and widely perceived as a drug associated with significant health and social risks, by users and non-users alike [61, 64–66]. Unfortunately, a large proportion of people who inject drugs (PWID) also experience significant stigma when engaging with healthcare providers [67]. Not only does this behavior discourage PWID from engaging with healthcare services while perpetuating negative attitudes within society, but also has detrimental economic implications for healthcare expenditure. For example, more than 5.5 million PWID are living with Hepatitis C which has accounted for almost half of the number of deaths associated with liver disease [2]. Reluctance to engage with health care services also contributes significantly to a lack of knowledge of blood-borne diseases among PWID. Inadequate knowledge of the health risks may also contribute to the initiation of drug injection [68]. Several studies have highlighted inadequate knowledge about sterilization practices required to prevent the transmission of blood-borne diseases among PWID [69, 70]. In a cohort of homeless individuals injecting drugs, limited awareness of the health risks was evident, as well as steps taken to mitigate risk given their limited circumstances [69]. It has also been shown that a small proportion of heroin users may combine heroin with other drugs which may also increase risks [68].

Awareness of the health risks and physiological effects associated with injecting drug use may prevent transitioning from smoking to injecting. A significant proportion of people who smoke heroin expressed a dislike for the intense physiological effects of injecting compared to smoking [66]. Alternatively, risk awareness may motivate some injecting heroin users to substitute heroin with a different drug [66]. Injecting heroin users cited awareness of health risks as a motivating factor to substitute heroin with cocaine, illicit methadone or other prescription opioids [71]. However, the latter substitution is particularly problematic as heroin users may overdose on these [72, 73].

There are also perceived risks related to factors external to injecting drug use, such as the risk of being arrested for possession, lack of affordability, and social isolation [68]. Interestingly, the social factors cited by heroin users that seem to be more influential in discouraging injection are negative observations made of friends, family, and/or partners [66]. Among a cohort of people who smoked heroin, with some occasionally injecting, significant concerns were highlighted as to the social implications of injecting versus smoking as injecting was deemed to pose risks of unemployment, relationship problems, and social isolation [66].

Conversely, social networks may also contribute to the initiation of heroin use with more than 70% of injecting drug users in Vietnam citing friends as the reason for first using heroin with a small proportion citing partners; most started by smoking heroin with very few starting with injecting [70]. Prolonged exposure to drug use has been highlighted as a potential risk factor in the escalation of drug use practices, from smoking to injecting heroin for example [70]. However, in a cohort of police detainees, the length and frequency of heroin use was associated with lowered risk perception, despite 30–35% of participants rating their local heroin market as very risky [74]. It has also been shown that PWID may also engage in other high-risk behaviors, either before or subsequent to initiating injecting drug use, with sex work being the main source of income for a significant proportion of PWID in Vietnam [70].

## Culture and tradition

Some drugs have persisted for thousands of years and continue to be used among communities and cultural groups as traditional herbs and remedies. For example, substances such as kratom, khat, kava, coca, ayahuasca, kambo, and peyote (mescaline) have been part of traditional cultural and religious practices for centuries in various parts of the world [75–77], but modern use in Western society is becoming increasingly popular. In Iran, there is a long history of opium use with a supportive culture that persists even today [11]. However, there are also examples of psychoactive plant-derived substances that have their origins in traditional use among local populations, where they have now been banned and diverted to the illicit trade within Western society. Contrastingly, peyote (mescaline) is classified as a Schedule I controlled substance in the US, with associated legal penalties for possession and sale [77]. However, members of the Native Americans Church are excepted because they ingest peyote legally during religious ceremony [77]. The cultural and traditional importance of these substances among some population groups introduces significant complexities in consideration and implementation of legal regulations relating to possession and use.

### Petrol/gasoline sniffing in indigenous communities

Volatile substance sniffing is a form of recreational drug use which involves the inhalation of certain chemical substances [78]. Petrol sniffing, an example of volatile substance misuse, is a persistent problem particularly among Indigenous communities in Australia [79]. The first accounts of petrol sniffing in Australia were from a Northern Territory in the 1940’s [80]. The appeal of petrol sniffing may lie in the rapid action of inhalants, the low cost, and the lack of access to other drugs [81]. Other reasons provided by individuals from an Aboriginal community cite rebellion, lack of parental control, and peer pressure [80]. Petrol sniffing among these communities has commenced at increasingly younger ages and appeared to be driven by psycho-social factors related to changes in social dynamics and even seasonal changes [82]. The most well-documented and successful intervention has been the introduction of low aromatic fuel which, while still harmful, does not produce the desired psychoactive effects due to reduced amounts of intoxicating solvents [79, 83]. The reasons why volatile substance misuse seems to be particularly prevalent among isolated indigenous communities, not only in Australia but in several other countries [84–86], is not yet completely understood.

## Conclusion

Accurate estimates of population drug use and an understanding of the factors that influence drug choice may assist in the development of appropriate and targeted prevention strategies. This review has highlighted several psychosocial and socioeconomic factors that influence drug use patterns for consideration in the development of future drug use interventions.

The last few decades have seen a shift in the profile of drug use in Australia, including the “phasing out” of traditional drugs in preference for newer, more popular alternatives. The phenomenon of the “substitution effect” has been observed in many contexts and should be considered in the development of legislation, particularly as governments transition to interventions governed by harm-minimization rather than legal penalties. Given the increasing popularity and availability of NPS, there is much uncertainty about how drug use patterns will evolve over the coming decades. Consideration of psychosocial factors (e.g. risk awareness and stigma) will also be important in developing approaches for harm minimization and subsequently, reducing associated morbidity and mortality. A knowledge of past, current, and emerging local drug use trends may assist health care professionals and public health agencies in understanding and evaluating the outcomes associated with newly emerging substances of abuse within populations.

## Key points

This paper:

- Provides a narrative review of socioeconomic and psychosocial determinants of substance misuse.
- Explores current and historical examples of substance misuse in Australia.
- Highlights the importance of understanding past and current trends in evaluating emerging substances of abuse.

## References

[1] Global Burden of Disease Collaborative Network. Global burden of disease study 2017. Seattle: Institute for Health Metrics and Evaluation; 2018.

[2] United Nations Office on Drugs and Crime. World Drug Report 2020. Austria: UNODC; 2020.

[3] Peacock A, Leung J, Larney S, Colledge S, Hickman M, Rehm J, Giovino GA, West R, Hall W, Griffiths P, Ali R, Gowing L, Marsden J, Ferrari AJ, Grebely J, Farrell M, Degenhardt L. Global statistics on alcohol, tobacco and illicit drug use: 2017 status report. Addiction. 2018;113:1905–26. 29749059 10.1111/add.14234

[4] Babor TF, Caulkins JP, Edwards G, Fischer B, Foxcroft DR, Humphreys K, Obot IS, Rehm J, Reuter P, Room R. Drug policy and the public good. Oxford: Oxford University Press; 2010.

[5] Australian Institute of Health and Welfare. Australian burden of disease study: impact and causes of illness and death in Australia 2018. Canberra: AIHW; 2021.

[6] Australian Institute of Health and Welfare. National drug strategy household survey 2019. Canberra: AIHW; 2020.

[7] Bicke WK, DeGrandpre RJ, Higgins ST, Hughes JR. Behavioral economics of drug self-administration. I. Functional equivalence of response requirement and drug dose. Life Sci. 1990;47:1501–10. 2250566 10.1016/0024-3205(90)90178-t

[8] Goldberg T. Will Swedish and Dutch drug policy converge? The role of theory. Int J Soc Welf. 2005;14:44–54.

[9] Colomer-Perez N, Chover-Sierra E, Navarro-Martinez R, Andriuseviciene V, Vlachou E, Cauli O. Alcohol and drug use in european university health science students: relationship with self-care ability*.* Int J Environ Res Public Health. 2019;16. 10.3390/ijerph16245042 PMC6949914 31835685

[10] Directorate-General for Communication. Flash Eurobarometer 401: young people and drugs. Belgium: European Commission; 2014.

[11] Momtazi S, Rawson R. Substance abuse among Iranian high school students. Curr Opin Psychiatry. 2010;23:221–6. 20308905 10.1097/YCO.0b013e328338630d PMC4479403

[12] Latypov A, Otiashvili D, Zule W. Drug scene, drug use and drug-related health consequences and responses in Kulob and Khorog. Tajikistan Int J Drug Policy. 2014;25:1204–14. 25449057 10.1016/j.drugpo.2014.09.011 PMC4294955

[13] Fast D, Small W, Krüsi A, Wood E, Kerr T. ‘I guess my own fancy screwed me over’: transitions in drug use and the context of choice among young people entrenched in an open drug scene. BMC Public Health. 2010;10:126. 20222984 10.1186/1471-2458-10-126 PMC2853507

[14] Delcher C, Bae J, Wang Y, Doung M, Fink DS, Young HW. Defining “doctor shopping”with dispensing data: a scoping review. Pain Med. 2022;23:1323–32. 34931686 10.1093/pm/pnab344

[15] Mars SG, Bourgois P, Karandinos G, Montero F, Ciccarone D. “Every ‘never’ I ever said came true”: transitions from opioid pills to heroin injecting. Int J Drug Policy. 2014;25:257–66. 24238956 10.1016/j.drugpo.2013.10.004 PMC3961517

[16] Reuter P, Trautmann F. A report of global illicit drug markets 1998–2007. Belgium: European Commission; 2009.

[17] United Nations Office on Drugs and Crime. World Drug Report 2019. Austria: UNODC; 2019.

[18] Van Buskirk J, Griffiths P, Farrell M, Degenhardt L. Trends in new psychoactive substances from surface and “dark” net monitoring. Lancet Psychiatry. 2017;4:16–8. 28012470 10.1016/S2215-0366(16)30405-9

[19] Moyle L, Childs A, Coomber R, Barratt MJ. #Drugsforsale: An exploration of the use of social media and encrypted messaging apps to supply and access drugs. Int J Drug Policy. 2019;63:101–10. 30530252 10.1016/j.drugpo.2018.08.005

[20] Wood JA. The Darknet: a digital copyright revolution. Richmond J Law Technol. 2010;16:14.

[21] Schwartz MJ. Feds bust ‘Farmer’s Market’ for online drugs. 2012. https://www.darkreading.com/attacks-and-breaches/feds-bust-farmers-market-for-online-drugs/d/d-id/1103901. Accessed 6 Oct 2020.

[22] Man N, Linghu Q, Bruno R, Sutherland R, Barratt M, Peacock A. Trends in the availability and types of drugs sold on the internet via cryptomarkets, October 2021 - September 2022. Sydney: National Drug and Alcohol Research Centre, UNSW Sydney; 2022.

[23] Van Buskirk J, Naicker S, Roxburgh A, Bruno R, Burns L. Who sells what? Country specific differences in substance availability on the Agora cryptomarket. Int J Drug Policy. 2016;35:16–23. 27520115 10.1016/j.drugpo.2016.07.004

[24] Australian Criminal Intelligence Commission. Illicit drug data report 2019–20. Canberra: ACIC; 2021.

[25] Demant J, Bakken SA, Oksanen A, Gunnlaugsson H. Drug dealing on Facebook, Snapchat and Instagram: a qualitative analysis of novel drug markets in the Nordic countries. Drug Alcohol Rev. 2019;38:377–85. 31050051 10.1111/dar.12932

[26] Degenhardt L, Hall W. Extent of illicit drug use and dependence, and their contribution to the global burden of disease. Lancet. 2012;379:55–70. 22225671 10.1016/S0140-6736(11)61138-0

[27] Benfer I, Zahnow R, Barratt MJ, Maier L, Winstock A, Ferris J. The impact of drug policy liberalisation on willingness to seek help for problem drug use: a comparison of 20 countries. Int J Drug Policy. 2018;56:162–75. 29731288 10.1016/j.drugpo.2018.03.032

[28] Australian Institute of Health and Welfare. National drug strategy household survey 2016. Canberra: AIHW; 2017.

[29] Vuolo M. National-level drug policy and young people’s illicit drug use: a multilevel analysis of the European Union. Drug Alcohol Depend. 2013;131:149–56. 23298650 10.1016/j.drugalcdep.2012.12.012

[30] United Nations Office on Drugs and Crime. World drug report 2022. Austria: UNODC; 2022.

[31] Martins SS, Mauro CM, Santaella-Tenorio J, Kim JH, Cerda M, Keyes KM, Hasin DS, Galea S, Wall M. State-level medical marijuana laws, marijuana use and perceived availability of marijuana among the general US population. Drug Alcohol Depend. 2016;169:26–32. 27755989 10.1016/j.drugalcdep.2016.10.004 PMC5140747

[32] Wen H, Hockenberry JM, Cummings JR. The effect of medical marijuana laws on adolescent and adult use of marijuana, alcohol, and other substances. J Health Econ. 2015;42:64–80. 25863001 10.1016/j.jhealeco.2015.03.007

[33] Hasin DS, Sarvet AL, Cerdá M, Keyes KM, Stohl M, Galea S, Wall MM. US adult illicit cannabis use, cannabis use disorder, and medical marijuana laws: 1991–1992 to 2012–2013. JAMA Psychiat. 2017;74:579–88. 10.1001/jamapsychiatry.2017.0724 PMC5539836 28445557

[34] Cerdá M, Mauro C, Hamilton A, Levy NS, Santaella-Tenorio J, Hasin D, Wall MM, Keyes KM, Martins SS. Association between recreational marijuana legalization in the United States and changes in marijuana use and cannabis use disorder from 2008 to 2016. JAMA Psychiat. 2020;77:165–71. 10.1001/jamapsychiatry.2019.3254 PMC6865220 31722000

[35] Compton WM, Han B, Jones CM, Blanco C, Hughes A. Marijuana use and use disorders in adults in the USA, 2002–14: analysis of annual cross-sectional surveys. Lancet Psychiatry. 2016;3:954–64. 27592339 10.1016/S2215-0366(16)30208-5

[36] Cerdá M, Wall M, Keyes KM, Galea S, Hasin D. Medical marijuana laws in 50 states: Investigating the relationship between state legalization of medical marijuana and marijuana use, abuse and dependence. Drug Alcohol Depend. 2012;120:22–7. 22099393 10.1016/j.drugalcdep.2011.06.011 PMC3251168

[37] Cerdá M, Wall M, Feng T, Keyes KM, Sarvet A, Schulenberg J, O’Malley PM, Pacula RL, Galea S, Hasin DS. Association of state recreational marijuana laws with adolescent marijuana use. JAMA Pediatr. 2017;171:142–9. 28027345 10.1001/jamapediatrics.2016.3624 PMC5365078

[38] Drug Enforcement Administration. Lists of: scheduling actions controlled substances regulated chemicals. 2023. https://www.deadiversion.usdoj.gov/schedules/orangebook/orangebook.pdf. Accessed 18 Aug 2023.

[39] Drug Enforcement Administration. Schedules of controlled substances; scheduling of 3,4-methylenedioxymethamphetamine (MDMA) into Schedule I of the Controlled Substances Act. Remand. 1988;53:5156.

[40] Australian Government. Drug laws in Australia. 2019. https://www.health.gov.au/topics/drugs/about-drugs/drug-laws-in-australia. Accessed 18 Aug 2023.

[41] Shapira B, Rosca P, Berkovitz R, Gorjaltsan I, Neumark Y. The switch from one substance-of-abuse to another: illicit drug substitution behaviors in a sample of high-risk drug users. PeerJ. 2020;8: e9461. 32742781 10.7717/peerj.9461 PMC7370931

[42] Potts AJ, Cano C, Thomas SHL, Hill SL. Synthetic cannabinoid receptor agonists: classification and nomenclature. Clin Toxicol (Phila). 2020;58:82–98. 31524007 10.1080/15563650.2019.1661425

[43] Byard RW, Gilbert J, James R, Lokan RJ. Amphetamine derivative fatalities in South Australia–is “Ecstasy” the culprit? Am J Forensic Med Pathol. 1998;19:261–5. 9760094 10.1097/00000433-199809000-00013

[44] González-Mariño I, Baz-Lomba JA, Alygizakis NA, Andrés-Costa MJ, Bade R, Bannwarth A, Barron LP, Been F, Benaglia L, Berset JD, Bijlsma L, Bodík I, Brenner A, Brock AL, Burgard DA, Castrignanò E, Celma A, Christophoridis CE, Covaci A, Delémont O, de Voogt P, Devault DA, Dias MJ, Emke E, Esseiva P, Fatta-Kassinos D, Fedorova G, Fytianos K, Gerber C, Grabic R, Gracia-Lor E, Grüner S, Gunnar T, Hapeshi E, Heath E, Helm B, Hernández F, Kankaanpaa A, Karolak S, Kasprzyk-Hordern B, Krizman-Matasic I, Lai FY, Lechowicz W, Lopes A, López de Alda M, López-García E, Löve ASC, Mastroianni N, McEneff GL, Montes R, Munro K, Nefau T, Oberacher H, O'Brien JW, Oertel R, Olafsdottir K, Picó Y, Plósz BG, Polesel F, Postigo C, Quintana JB, Ramin P, Reid MJ, Rice J, Rodil R, Salgueiro-González N, Schubert S, Senta I, Simões SM, Sremacki MM, Styszko K, Terzic S, Thomaidis NS, Thomas KV, Tscharke BJ, Udrisard R, van Nuijs ALN, Yargeau V, Zuccato E, Castiglioni S, Ort C. Spatio-temporal assessment of illicit drug use at large scale: evidence from 7 years of international wastewater monitoring*.* Addiction. 2020;115:109–120. 10.1111/add.14767 PMC6973045 31642141

[45] Lewis D, Kenneally M, van den Heuvel C, Byard RW. Methamphetamine deaths: changing trends and diagnostic issues. Med Sci Law. 2021;61:130–7. 33423599 10.1177/0025802420986707

[46] Lewis D, Kenneally M, van den Heuvel C, Byard RW. Increasing age and methamphetamine use. J Forensic Leg Med. 2021;80: 102181. 33991928 10.1016/j.jflm.2021.102181

[47] Australian Criminal Intelligence Commission. Illicit Drug Data Report 2017–18. Canberra: ACIC; 2019.

[48] Australian Criminal Intelligence Commission. Illicit Drug Data Report 2014–15. Canberra: ACIC; 2016.

[49] Roche A, McEntee A. Ice and the outback: patterns and prevalence of methamphetamine use in rural Australia. Aust J Rural Health. 2017;25:200–9. 27868256 10.1111/ajr.12331

[50] Sutherland R, Uporova J, King C, Jones F, Karlsson A, Gibbs D, Price O, Bruno R, Dietze P, Lenton S, Salom C, Daly C, Thomas N, Juckel J, Agramunt S, Wilson Y, Que Noy W, Wilson J, Degenhardt L, Farrell M, Peacock A. Australian Drug Trends 2022: key findings from the national illicit drug reporting system (IDRS) interviews. Sydney: National Drug and Alcohol Research Centre, UNSW Sydney; 2022.

[51] Van Buskirk J, Roxburgh A, Bruno R, Naicker S, Lenton S, Sutherland R, Whittaker E, Sindicich N, Matthews A, Butler K, Burns L. Characterising dark net marketplace purchasers in a sample of regular psychostimulant users. Int J Drug Policy. 2016;35:32–7. 26872846 10.1016/j.drugpo.2016.01.010

[52] Mars SG, Rosenblum D, Ciccarone D. Illicit fentanyls in the opioid street market: desired or imposed? Addiction. 2019;114:774–80. 30512204 10.1111/add.14474 PMC6548693

[53] Deering KN, Shoveller J, Tyndall MW, Montaner JS, Shannon K. The street cost of drugs and drug use patterns: relationships with sex work income in an urban Canadian setting. Drug Alcohol Depend. 2011;118:430–6. 21704461 10.1016/j.drugalcdep.2011.05.005 PMC3392208

[54] Australian Crime Commission. Illicit drug data report 2005–06. Canberra: ACC; 2007.

[55] Australian Crime Commission. Illicit drug data report 2010–11. Canberra: ACC; 2012.

[56] Tosevski DL, Milovancevic MP, Gajic SD. Personality and psychopathology of university students. Curr Opin Psychiatry. 2010;23:48–52. 19890212 10.1097/YCO.0b013e328333d625

[57] Gupta S, Sarpal SS, Kumar D, Kaur T, Arora S. Prevalence, pattern and familial effects of substance use among the male college students -a north Indian study. J Clin Diagn Res. 2013;7:1632–6. 24086860 10.7860/JCDR/2013/6441.3215 PMC3782917

[58] Lai FY, O’Brien J, Bruno R, Hall W, Prichard J, Kirkbride P, Gartner C, Thai P, Carter S, Lloyd B, Burns L, Mueller J. Spatial variations in the consumption of illicit stimulant drugs across Australia: A nationwide application of wastewater-based epidemiology. Sci Total Environ. 2016;568:810–8. 27267725 10.1016/j.scitotenv.2016.05.207

[59] Yi S, Peltzer K, Pengpid S, Susilowati IH. Prevalence and associated factors of illicit drug use among university students in the association of southeast Asian nations (ASEAN). Subst Abuse Treat Prev Policy. 2017;12:9. 28381234 10.1186/s13011-017-0096-3 PMC5382470

[60] Man N, Chrzanowska A, Price O, Bruno R, Dietze PM, Sisson SA, Degenhardt L, Salom C, Morris L, Farrell M, Peacock A. Trends in cocaine use, markets and harms in Australia, 2003–2019. Drug Alcohol Rev. 2021;40:946–56. 33626201 10.1111/dar.13252

[61] Lipari RN, Ahrnsbrak RD, Pemberton MR, Porter JD. Risk and protective factors and estimates of substance use initiation: results from the 2016 national survey on drug use and health. In: NSDUH data review. Rockville (MD): Substance Abuse and Mental Health Services Administration; 2017. 29431965

[62] Holt LJ, Looby A. Factors that differentiate prescription stimulant misusers from those at-risk for misuse: expectancies, perceived safety, and diversion. Subst Use Misuse. 2018;53:1068–75. 29220608 10.1080/10826084.2017.1392984

[63] Fox J, Smith A, Yale A, Chow C, Alaswad E, Cushing T, Monte AA. Drugs of abuse and novel psychoactive substances at outdoor music festivals in Colorado. Subst Use Misuse. 2018;53:1203–11. 29148866 10.1080/10826084.2017.1400067 PMC5935531

[64] Cheeta S, Halil A, Kenny M, Sheehan E, Zamyadi R, Williams AL, Webb L. Does perception of drug-related harm change with age? A cross-sectional online survey of young and older people. BMJ Open. 2018;8: e021109. 30401725 10.1136/bmjopen-2017-021109 PMC6231571

[65] Brown SA. Stigma towards marijuana users and heroin users. J Psychoact Drugs. 2015;47:213–20. 10.1080/02791072.2015.1056891 26148124

[66] Harris J, Shorter GW, Davidson G, Best P. Risk perception, changing social context, and norms prevent transition to regular injection among people who smoke heroin. Drug Alcohol Depend. 2020;208: 107878. 32014646 10.1016/j.drugalcdep.2020.107878

[67] Biancarelli DL, Biello KB, Childs E, Drainoni M, Salhaney P, Edeza A, Mimiaga MJ, Saitz R, Bazzi AR. Strategies used by people who inject drugs to avoid stigma in healthcare settings. Drug Alcohol Depend. 2019;198:80–6. 30884432 10.1016/j.drugalcdep.2019.01.037 PMC6521691

[68] White G, Luczak SE, Mundia B, Goorah S. Exploring the perceived risks and benefits of heroin use among young people (18–24 years) in Mauritius: economic insights from an exploratory qualitative study. Int J Environ Res Public Health. 2020;17:6126. 32842510 10.3390/ijerph17176126 PMC7503563

[69] Wright NM, Tompkins CN, Jones L. Exploring risk perception and behaviour of homeless injecting drug users diagnosed with hepatitis C. Health Soc Care Community. 2005;13:75–83. 15717909 10.1111/j.1365-2524.2005.00552.x

[70] Khuat OT, Morrow M, Nguyen TN, Armstrong G. Social context, diversity and risk among women who inject drugs in Vietnam: descriptive findings from a cross-sectional survey. Harm Reduct J. 2015;12:35. 26472467 10.1186/s12954-015-0067-9 PMC4608123

[71] Shapira B, Berkovitz R, Rosca P, Lev-Ran S, Kaptsan A, Neumark Y. Why Switch? - Motivations for Self-Substitution of Illegal Drugs. Subst Use Misuse. 2021;56:627–38. 33663337 10.1080/10826084.2021.1887246

[72] Fomin D, Baranauskaite V, Usaviciene E, Sumkovskaja A, Laima S, Jasulaitis A, Minkuviene ZN, Chmieliauskas S, Stasiuniene J. Human deaths from drug overdoses with carfentanyl involvement—new rising problem in forensic medicine: a STROBE-compliant retrospective study. Medicine. 2018;97: e13449. 30508965 10.1097/MD.0000000000013449 PMC6283219

[73] Misailidi N, Papoutsis I, Nikolaou P, Dona A, Spiliopoulou C, Athanaselis S. Fentanyls continue to replace heroin in the drug arena: the cases of ocfentanil and carfentanil. Forensic Toxicol. 2018;36:12–32. 29367860 10.1007/s11419-017-0379-4 PMC5754389

[74] Payne JL, Langfield CT. How risky are heroin markets? A multi-site study of self-reported risk perceptions among police detainees in Australia. Int J Drug Policy. 2020;90: 103062. 33348184 10.1016/j.drugpo.2020.103062

[75] Dos Santos R, Bouso JC, Hallak J. Ayahuasca: what mental health professionals need to know*.* Arch Clin Psychiatry. 2017;44.

[76] Sacco MA, Zibetti A, Bonetta CF, Scalise C, Abenavoli L, Guarna F, Gratteri S, Ricci P, Aquila I. Kambo: Natural drug or potential toxic agent? A literature review of acute poisoning cases. Toxicol Rep. 2022;9:905–13. 35515815 10.1016/j.toxrep.2022.04.005 PMC9061256

[77] Dasgupta A, Wahed A. Chapter 17 - challenges in drugs of abuse testing: magic mushrooms, peyote cactus, and designer drugs. In: Dasgupta A, Wahed A, editors. Clinical chemistry, immunology and laboratory quality control. San Diego: Elsevier; 2014. p. 307–16.

[78] Dell CA, Gust SW, MacLean S. Global issues in volatile substance misuse. Subst Use Misuse. 2011;46(Suppl 1):1–7. 21609139 10.3109/10826084.2011.580169

[79] d’Abbs P, Shaw G, Field E. The impact of subsidized low aromatic fuel (LAF) on petrol (gasoline) sniffing in remote Australian indigenous communities. Subst Abuse Treat Prev Policy. 2017;12:38. 28818114 10.1186/s13011-017-0121-6 PMC5561594

[80] Senior K, Chenhall R, Daniels D. “Stuck Nose”: Experiences and understanding of petrol sniffing in a remote aboriginal community. Contemp Drug Probl. 2006;33:451–72.

[81] Byard RW, Chivell WC, Gilbert JD. Unusual facial markings and lethal mechanisms in a series of gasoline inhalation deaths. Am J Forensic Med Pathol. 2003;24:298–302. 12960669 10.1097/01.paf.0000083548.52978.40

[82] Garrow A. Time to stop reinventing the wheel: petrol sniffing in the top end project final report. Darwin: Alcohol and Other Drugs Program, Territory Health Services; 1997.

[83] Stephenson L, Grabowski M, van den Heuvel C, Humphries M, Byard RW. Success of low aromatic fuel in preventing gasoline sniffing deaths. Am J Forensic Med Pathol. 2022;43:354–8. 35970515 10.1097/PAF.0000000000000786

[84] Coleman H, Charles G, Collins J. Inhalant use by Canadian Aboriginal youth. J Child Adolesc Subst Abuse. 2001;10:1–20.

[85] Beauvais F, Wayman JC, Jumper-Thurman P, Plested B, Helm H. Inhalant abuse among American Indian, Mexican American, and non-Latino white adolescents. Am J Drug Alcohol Abuse. 2002;28:171–87. 11853132 10.1081/ada-120001287

[86] Zebrowski PL, Gregory RJ. Inhalant use patterns among Eskimo school children in western Alaska. J Addict Dis. 1996;15:67–77. 8842851 10.1300/J069v15n03_05

[87] Australian Crime Commission. Illicit Drug Data Report 2002–03. Canberra: ACC; 2004.

[88] Australian Crime Commission. Illicit Drug Data Report 2003–04. Canberra: ACC; 2005.

[89] Australian Crime Commission. Illicit Drug Data Report 2004–05. Canberra: ACC; 2006.

[90] Australian Crime Commission. Illicit Drug Data Report 2006–07 (Revised Edition). Canberra: ACC; 2009.

[91] Australian Crime Commission. Illicit Drug Data Report 2007–08. Canberra: ACC; 2009.

[92] Australian Crime Commission. Illicit Drug Data Report 2008–09. Canberra: ACC; 2010.

[93] Australian Crime Commission. Illicit Drug Data Report 2009–10. Canberra: ACC; 2011.

[94] Australian Crime Commission. Illicit Drug Data Report 2011–12. Canberra: ACC; 2013.

[95] Australian Crime Commission. Illicit Drug Data Report 2012–13. Canberra: ACC; 2014.

[96] Australian Crime Commission. Illicit Drug Data Report 2013–14. Canberra: ACC; 2015.

[97] Australian Criminal Intelligence Commission. Illicit Drug Data Report 2015–16. Canberra: ACIC; 2017.

[98] Australian Criminal Intelligence Commission. Illicit Drug Data Report 2016–17. Canberra: ACIC; 2018.

[99] Australian Criminal Intelligence Commission. Illicit Drug Data Report 2018–19. Canberra: ACIC; 2020.

